# Quasi‐Parallel Shock Reformation Seen by Magnetospheric Multiscale and Ion‐Kinetic Simulations

**DOI:** 10.1029/2021GL096335

**Published:** 2022-01-25

**Authors:** Andreas Johlander, Markus Battarbee, Lucile Turc, Urs Ganse, Yann Pfau‐Kempf, Maxime Grandin, Jonas Suni, Vertti Tarvus, Maarja Bussov, Hongyang Zhou, Markku Alho, Maxime Dubart, Harriet George, Konstantinos Papadakis, Minna Palmroth

**Affiliations:** ^1^ Swedish Institute of Space Physics Uppsala Sweden; ^2^ Department of Physics University of Helsinki Helsinki Finland; ^3^ Finnish Meteorological Institute Helsinki Finland

**Keywords:** collisionless shock, quasi‐parallel, shock reformation, satellite measurements, plasma simulation

## Abstract

Shock waves in collisionless plasmas are among the most efficient particle accelerators in space. Shock reformation is a process important to plasma heating and acceleration, but direct observations of reformation at quasi‐parallel shocks have been lacking. Here, we investigate Earth's quasi‐parallel bow shock with observations by the four Magnetospheric Multiscale spacecraft. The multi‐spacecraft observations provide evidence of short large‐amplitude magnetic structures (SLAMS) causing reformation of the quasi‐parallel shock. We perform an ion‐kinetic Vlasiator simulation of the bow shock and show that SLAMS reforming the bow shock recreates the multi‐spacecraft measurements. This provides a method for identifying shock reformation in the future.

## Key Points

- We present observations of the four MMS spacecraft crossing Earth's bow shock seemingly in the reverse order
- The observations, supported by an ion‐kinetic simulation, reveal that short‐large amplitude magnetic structures cause the shock to reform
- These multispacecraft signatures can be used in the future to identify shock reformation

## 1 Introduction

Collisionless shock waves are some of the most energetic phenomena in astrophysical plasmas. Shock waves form around planets, stars, galaxies, and supernova remnants where they are responsible for the generation of galactic cosmic rays through diffusive shock acceleration (e.g., Abdo et al., 2010; Bell, 1978). A characteristic feature of shocks is their ability to reflect a portion of the incoming ions back into the upstream plasma. The angle *θ*_Bn_ between the shock normal and the upstream magnetic field then determines the structure of the shock. In quasi‐perpendicular shocks, where *θ*_Bn_ ≳ 45°, reflected ions are turned around by the upstream magnetic field (Sckopke et al., 1983) and the shock appears as a sharp transition between upstream and downstream. If the shock instead is quasi‐parallel with *θ*_Bn_ ≲ 45°, reflected ions travel upstream, exciting waves and turbulence and forming the foreshock. This causes the quasi‐parallel shock transition to be more extended and dynamic (Schwartz & Burgess, 1991).

Early observations (Greenstadt, 1985) from Earth's bow shock and simulations (Burgess, 1989; Thomas et al., 1990) found that the quasi‐parallel shock can be nonstationary, meaning that the structure of the shock changes periodically in time. An important type of structure in the quasi‐parallel shock are upstream shock‐like pulsations known as short large‐amplitude magnetic structures (SLAMS; Schwartz & Burgess, 1991). SLAMS propagate against the flow in the plasma frame but are advected toward the shock (Schwartz et al., 1992) and they are thought to form from ultra‐low frequency (ULF) waves (Thomsen et al., 1988). Proposed formation mechanisms for SLAMS involve buildup of energetic ion pressure on the downstream edge of the SLAMS (Giacalone et al., 1993) and a nonresonant instability (Bell, 2004) of the ULF waves with backstreaming ions (Chen et al., 2021). While Earth's quasi‐parallel bow shock has been described as a wide patchwork of SLAMS (Schwartz & Burgess, 1991), simulations often show a less extended transition where SLAMS form closer to the shock and participate in the cyclical reformation of the shock (e.g., Burgess, 1995; Caprioli et al., 2015; Hao et al., 2017; Scholer et al., 2003; Tsubouchi & Lembège, 2004). Simulations by Scholer (1993) showed that quasi‐parallel reformation is dominated by SLAMS that steepen ∼20 inertial lengths upstream of the shock. Using observations from the four Cluster satellites, Lucek et al. (2008) found that SLAMS inhabit a rather narrow region (few thousand kilometers) upstream of the shock. SLAMS have long been considered to be important for the variability and reformation of quasi‐parallel shocks, but direct observations have so far been lacking.

There has been recent advancement on shock reformation with the high‐cadence measurements from the Magnetospheric Multiscale (MMS) spacecraft. Yang et al. (2020) found evidence of shock reformation of the quasi‐perpendicular bow shock and Turner et al. (2021) found evidence for ion‐kinetic scale reformation of a shock upstream of a hot flow anomaly. Madanian et al. (2021) investigated a quasi‐perpendicular high‐Mach number shock (*M*_*A*_ ∼ 27) with periodic behavior, similar to those observed at Saturn's bow shock (Sulaiman et al., 2015), and found signatures of both shock rippling (Winske & Quest, 1988) and reformation. Liu et al. (2021) used an oblique (*θ*_Bn_ ∼ 50°) crossing from when MMS was in a string‐of‐pearls formation and showed that upstream ULF waves in the foreshock cause a cyclical reformation of the shock. They also found that ion reflection is modulated by the reformation and that the remnants of the old shock ramps could be observed in the downstream. Despite these recent advancements, it is still not clear how reformation manifests itself in quasi‐parallel shocks and how upstream waves and pulsations interact with the shock.

In this article, we present direct observational evidence of SLAMS participating in quasi‐parallel shock reformation. We use multipoint observational data from the MMS satellites during a crossing of Earth's bow shock. We further demonstrate, using a global hybrid‐Vlasov simulation, how shock reformation by SLAMS recreates the observed multi‐spacecraft signatures.

## 2 Observations

We use data from the four MMS spacecraft (Burch et al., 2016). Magnetic field data are from the fluxgate magnetometer (Russell et al., 2016) which collects the magnetic field vector with a cadence of 128 Hz. Since the full resolution data from MMS4 are not available at this time, we use the survey mode data which are collected with a cadence of 8 Hz for this spacecraft. The ion data are from the fast plasma investigation dual ion spectrometer (Pollock et al., 2016) instrument, which measures the ion distributions and moments every 150 ms. To avoid sampling the disturbances in the immediate upstream foreshock region, we obtain the asymptotic upstream field and plasma values from the Wind spacecraft (Lepping et al., 1995; Ogilvie et al., 1995), situated upstream of Earth. The Wind data, time‐shifted to the bow shock, are obtained from the OMNI database (King & Papitashvili, 2005).

**Figure 1:**
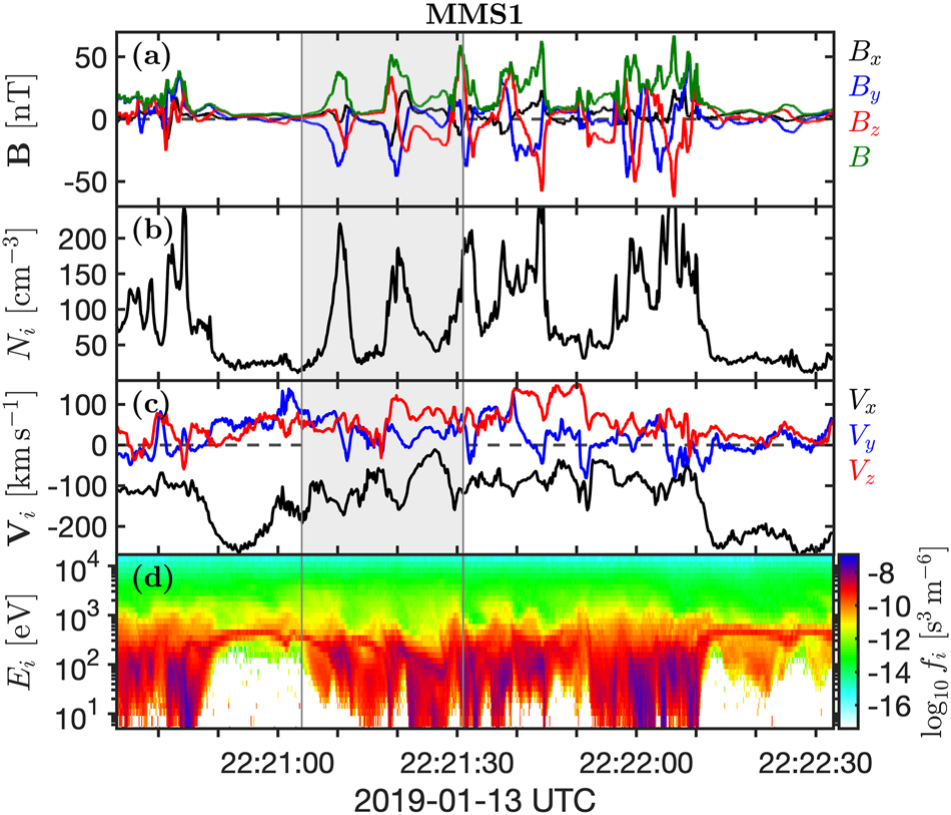
MMS1 observations of a quasi‐parallel shock crossing. (a) Magnetic field in GSE. (b) Ion number density. (c) Ion bulk velocity in GSE. (d) Omnidirectional ion phase‐space density as a function of energy.

Figure 1 shows a series of quasi‐parallel shock crossings by MMS1 on 13 January 2019 around 22:21 UTC. The spacecraft were on the outward leg of their orbit, positioned at ∼(11, −3, 5)*R*_*E*_ in Geocentric Solar Ecliptic (GSE) coordinates, where *R*_*E*_ = 6371.2 km is the Earth radius. MMS was in a tetrahedron formation with spacecraft separation of ∼32 km and the solar wind was characterized by a relatively slow speed and high number density, see Table 1. MMS start out in the downstream magnetosheath plasma and the shock motion causes the spacecraft to cross to the upstream foreshock, evident by the presence of the cold solar wind beam around 22:21:00 in Figure 1d. The bow shock motion then reverses to the sunward direction and overtakes the spacecraft orbital motion (Maksimovic et al., 2003), leading them to cross back to the downstream plasma in an extended crossing marked with gray in Figure 1. During this crossing, MMS observe a number of large compressions of the magnetic field magnitude *B* and ion number density *N*_*i*_, indicating the presence of SLAMS. The *B*‐amplitude of the SLAMS increases as MMS gets closer to the shock and proceed into the downstream, consistent with previous observations (Lucek et al., 2008). From the large‐amplitude compressive structures, we conclude that the spacecraft stay relatively close to the bow shock. However, it is evident from the large density compression and significantly heated solar wind that the spacecraft is located in a shocked downstream plasma. The spacecraft then return to the upstream foreshock around 22:22:10.

**Table 1:** Upstream and Shock Parameters at the Spacecraft Location in the MMS Observations and in the Vlasiator Simulation

Parameter	Observations	Simulation
Upstream number density *N*_*u*_	22 cm^−3^	1 cm^−3^
Bulk speed *V*_*u*_	300 km s^−1^	750 km s^−1^
Magnetic field **B**_*u*_	(3.7,‐2.8,1.6) nT	(−2.99,0.26,0) nT
Shock normal vector $\hat{n}$	(0.96,‐0.15,0.22)	(0.97,0.24)
Shock angle *θ*_Bn_	∼30°	19°
Alfvén Mach number *M*_*A*_	13	11
Fast mode Mach *M*_*f*_	8	9
Upstream ion *β*_*i*,*u*_	0.5	1.9
Ion inertial length *d*_*i*,*u*_	48 km	230 km
Ion gyroperiod *T*_*ci*,*u*_	13 s	22 s
SLAMS leading edge speed	100 km s^−1^	380 km s^−1^

*Note*. Vector quantities are in GSE coordinates.

An important parameter for our analysis is the angle *θ*_Bn_ between the upstream magnetic field **B**_*u*_ and the shock normal vector $\hat{n}$. To determine $\hat{n}$, we use the bow shock model by Farris et al. (1991) fitted to the position of MMS (Schwartz, 1998). We find that the shock Alfvén Mach number is 13 and *θ*_Bn_ is around 30°, which means this is a quasi‐parallel shock. We validate the upstream solar wind measurements from Wind with local values obtained by MMS in the foreshock after 22:22:10 and find a good match between the measurements. There is still some uncertainty in *θ*_Bn_ because of the uncertainty of the time‐shift of the solar wind and because the bow shock itself can have large‐scale rippling causing $\hat{n}$ to fluctuate and *θ*_Bn_ to change during the event (Hao et al., 2016, 2017).

**Figure 2:**
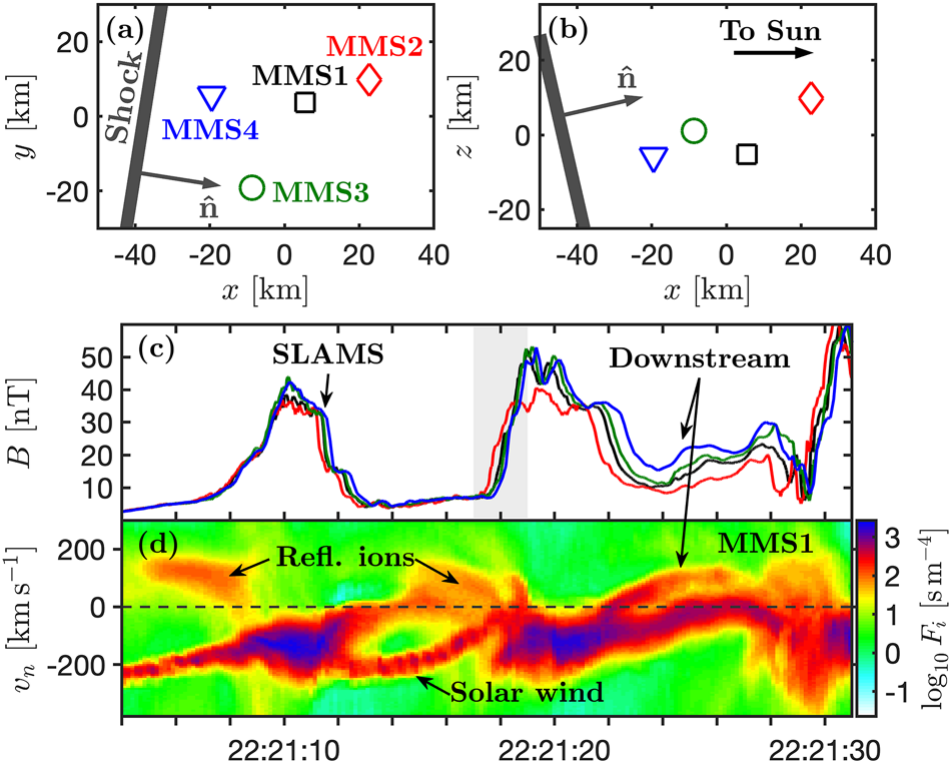
Four‐spacecraft observation of the shock transition from the shaded interval in Figures 1 (a) and 1(b) Spacecraft positions relative to the tetrahedron center in the GSE *x*‐*y* and *x*‐*z* planes. The shock orientation and $\hat{n}$ before the shock crossing are shown. (c) *B* observed by the four spacecraft. (d) Reduced ion distribution as a function of *v*_*n*_ observed by MMS1.

Figure 2 shows the relative positions of the four spacecraft and a zoomed‐in interval of the shock crossing. To more clearly visualize the ion distribution function, we show the reduced ion phase‐space density *F*_*i*_ as a function of velocity along $\hat{n}$ in Figure 2d. We see that there is a relatively clear distinction between times where MMS1 observes the cold solar wind beam, that may be decelerated, and where this beam is heated and compressed significantly. We note, however, that both these types of plasmas are in the extended shock transition region.

Figure 2c shows that the first sharp increase in *B* is advected downstream across the spacecraft and that MMS1 returns to the upstream where it again observes the cold solar wind beam. Since this structure reaches a value of *B*/*B*_*u*_ ∼ 8, we conclude that this is a SLAMS situated upstream of the bow shock. We see that there is a reflected ion population with *v*_*n*_ ≳ 0 just before the SLAMS (also reported by Chen et al. (2021)). Since the SLAMS is moving antisunward in the spacecraft frame, these reflected ions are antisunward of the SLAMS and moving toward it. The same type of reflected ion population is seen after the SLAMS and before the next structure. The second sharp increase in *B* appears slightly different to the first SLAMS since MMS1 does not observe the cold solar wind beam again directly after the encounter. Instead, the spacecraft observes heated and compressed plasma even after the peak in *B*. This increase in *B* can appear at first glance to be the shock ramp proper that MMS crosses from the upstream to downstream. But, as we shall see, this is not the case.

Figure 2c shows that the first spacecraft to observe the increase in *B* is MMS2, followed by MMS1, MMS3, and finally MMS4. This is the reverse order of what would be expected of an upstream‐to‐downstream shock crossing since, as we see in Figures 2a and 2b, MMS2 is the spacecraft positioned furthest upstream and the other spacecraft are further downstream. So, while the overall shock motion is directed upstream, the motion of this structure is directed downstream. This means that what appears to be the shock crossing is, in fact, a SLAMS that is merging with the shock. Throughout the event in Figure 2, there is no conventional shock crossing where the shock simply moves along $\hat{n}$ over the four spacecraft. Instead a train of SLAMS merges with the bow shock, causing the shock to jump forward, leading to the spacecraft ending up in the heated and compressed downstream plasma.

To quantify the motion of the merging SLAMS, we perform a four‐spacecraft timing analysis. We find the time delays by minimizing the mean square deviation of *B* in the shaded area in Figure 2c. From this, we find that the velocity of the leading edge of the structure is 100 km s^−1^ and the direction of propagation is (−0.64, −0.05, −0.76), which is 140° from $\hat{n}$. Slightly different *B*‐profiles seen by the spacecraft indicate non‐planarity or evolution of the structure. Error analysis (Vogt et al., 2011) shows that the uncertainty in propagation speed is ∼20 km s^−1^ and the uncertainty in direction is ∼15°. This means that the structure is moving with a small angle toward the shock with a speed significantly lower than the solar wind, meaning that the structure is propagating against the flow in the plasma frame. We find that the trailing edge moves downstream at ∼34 km s^−1^, meaning that the SLAMS has slowed down as it merged with the shock. The timing analysis confirms that what appears to be a shock crossing is instead the downstream edge of a SLAMS impacting the shock.

## 3 Simulation

To validate and to investigate the scales of the SLAMS reformation process, we perform a two‐dimensional simulation of the bow shock with the global hybrid‐Vlasov code Vlasiator (Palmroth et al., 2018; von Alfthan et al., 2014). In the simulation, ions are described as distribution functions which are propagated with the Vlasov equation and electrons are modeled as an adiabatic charge‐neutralizing fluid. The electric field is closed by the generalized Ohm's law $E=-V_{i}\times B+\left(j\times B-\nabla \cdot P_{e}\right)/N_{i}q_{e}$ where **j** is the current density, *q*_*e*_ is the elementary charge, and $P_{e}$ is the electron pressure tensor, using isotropic and adiabatic electron heating. The simulation provides a global view of the quasi‐parallel bow shock and allows us to investigate scales not achievable with the small spacecraft separation of MMS.

**Figure 3:**
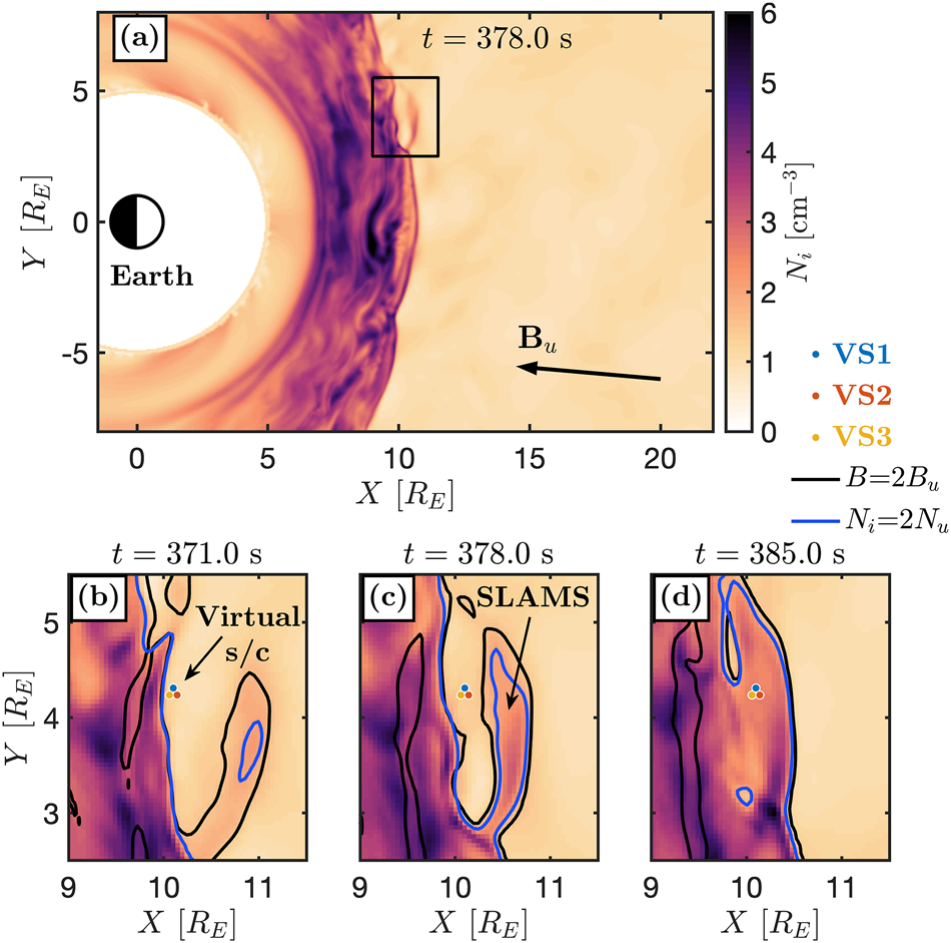
Vlasiator simulation of Earth's bow shock. (a) Part of the simulation showing *N*_*i*_. (b)–(d) Zoomed‐in snapshots in the simulation at different times. The three virtual spacecraft are shown as colored dots. Black (blue) contour line shows where *B* (*N*_*i*_) is two times the upstream value.

The simulation box is in the GSE *X*‐*Y* plane with *X* ∈ [−8, 72]*R*_*E*_ and *Y* ∈ [−32, 32]*R*_*E*_ with a grid resolution of 300 km and a velocity grid resolution of 30 km s^−1^. The inner boundary at 5*R*_*E*_ is a nearly perfect conductor. The solar wind flows from the upstream edge in the negative *X*‐direction. The upstream solar wind conditions of the simulation can be seen in Table 1. Figure 3a shows a part of the simulation box at the time *t* = 378 s where we see the curved, quasi‐parallel, bow shock as an increase in *N*_*i*_. See the Supporting Information for an animation of a part of the simulation.

We would like to preface the simulation analysis with discussion on the solar wind parameters in Table 1. First, the relatively fast solar wind speed is used so that fewer computational resources are spent while the bow shock initializes. Second, the relatively low solar wind density of 1 cm^−3^ and magnetic field of 3 nT are set in part to approximately match the observed *M*_*A*_ and in part to keep *d*_*i*,*u*_ comparable to the grid resolution. The local shock parameters that determine most of the relevant physics at the shock are similar to those observed during the shock crossing by MMS. However, *β*_*i*,*u*_ is higher in the simulation because the uniform velocity grid resolution requires a certain temperature to resolve the solar wind well without increasing the computational cost of the remaining velocity domain excessively.

Several SLAMS‐like structures form and merge with the shock during the simulation. The timescales on which SLAMS are seen here are slower than in Figure 2, likely due to the greater ion gyroperiod in the simulation. We can see an example of a SLAMS just upstream of the bow shock at *X* ∼ 10*R*_*E*_ and *Y* ∼ 4*R*_*E*_ in Figure 3a, marked with a black box. To obtain $\hat{n}$ of the shock at the position of the SLAMS, we use a fourth‐order polynomial fit to where *N*_*i*_ = 2*N*_*u*_ (Battarbee et al., 2020) and determine the normal to that curve. The resulting $\hat{n}$ and the shock parameters are shown in Table 1. The parameters *M*_*A*_ = 11 and *θ*_Bn_ = 19° are similar to those observed by MMS, despite the different solar wind conditions.

To better interpret the MMS multipoint measurements, we insert three *virtual spacecraft* into the simulation. The virtual spacecraft sample the field and plasma parameters to create time series that mimic spacecraft observations. The large scale bow shock motion is naturally directed sunward due to the two‐dimensional simulation setup and constant solar wind conditions. The spacecraft are positioned in an equilateral triangle centered around a simulation cell where the ion distribution function is saved. The plasma quantities are interpolated to the position of the virtual spacecraft. The inter‐spacecraft distance is ∼520 km which corresponds to 2.3*d*_*i*,*u*_.

Figures 3b–3d show three snapshots of *N*_*i*_ at the bow shock in the simulation with the virtual spacecraft (denoted VS1‐3) positions indicated. At *t* = 371 s, there is a magnetic structure visible upstream of the spacecraft. This structure is ∼1*R*_*E*_ (∼30*d*_*i*,*u*_) upstream of the shock surface and has enhanced *B* and *N*_*i*_ above two times their upstream values. The spatial extent of the structure is on the order of ∼1,000 km normal to the shock and ∼1*R*_*E*_ perpendicular to the shock. At *t* = 378 s, this structure has further grown in amplitude and has a maximum value of *B* more than four times the upstream value. This structure is therefore similar to the SLAMS observed by MMS. At *t* = 385 s, the SLAMS has encountered the shock, causing it to jump forward over the spacecraft, which are now positioned downstream of the shock. The spacecraft later cross back to the upstream at *t* ∼ 420 s similar to MMS in the previous section. At the same time, another SLAMS is forming upstream of the spacecraft, see the supporting animation.

**Figure 4:**
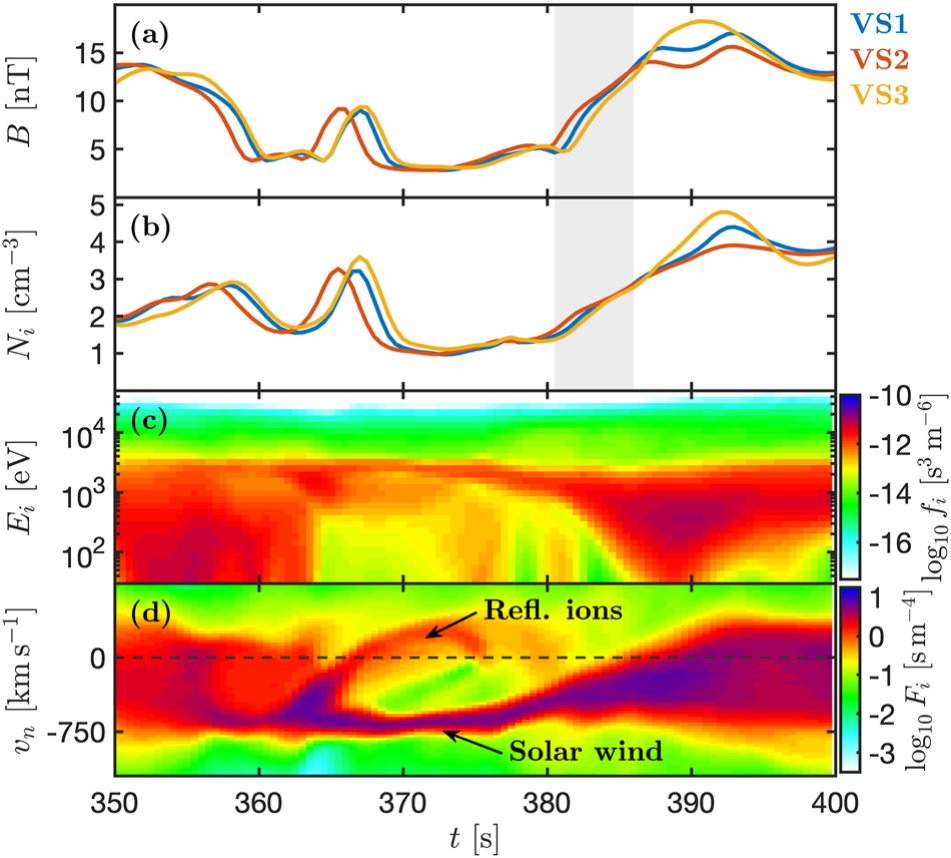
Multipoint virtual spacecraft observations in the Vlasiator simulation. (a) (*B*) (b) *N*_*i*_. (c) Omnidirectional ion phase‐space density. (d) Reduced ion distribution as a function of *v*_*n*_.

Next, we look at the time series “measurementsE” made by the virtual spacecraft, shown in Figure 4. The spacecraft start out downstream of the shock and then cross to the upstream at *t* ∼ 368 s. The spacecraft then observe the cold solar wind beam and a reflected ion population simultaneously at ∼375 s, similar to the MMS observations, see Figure 2d. The spacecraft then observe the SLAMS as a peak in *B* with *B*/*B*_*u*_ ∼ 6. Like in the observations, the spacecraft continue to observe heated and compressed plasma even after the SLAMS has merged with the shock, as can be seen in Figures 4c and 4d. We note that the trailing edge of the SLAMS, seen in the downstream plasma, is less pronounced than in the MMS observations. However, we see in the supporting animation after ∼392 s that there is a compressive structure moving antisunward in the magnetosheath, making the exact downstream signatures sensitive to where the virtual spacecraft are placed.

Despite only observing one SLAMS, the multi‐spacecraft time series in the simulation show the same signature as the MMS observations at the shock transition. We see in Figure 4a that VS2, which is the spacecraft furthest upstream (see Figures 3b–3d), observes the increase in *B* first, followed by VS1 and then VS3. This shows that, while the overall shock motion is directed upstream, reformation by a SLAMS leads to the same reverse order in *B* as in the MMS observations. We perform a timing analysis of this structure, which is applicable using three spacecraft instead of four because of the two‐dimensional geometry of the simulation. Using the measured *B* in the gray interval in Figure 4a, we find that the structure is propagating with 380 km s^−1^ along (−1.00, 0.07) which is 160° away from $\hat{n}$. This means that like in the observations, the structure is moving with a small angle toward the shock and is propagating against the flow in the plasma frame. These observations confirm that a SLAMS which is impacting the shock, causing it to reform, does result in the multipoint observational signatures seen by MMS.

## 4 Conclusions

In this work, we report the first direct evidence of SLAMS participating in the shock reformation process of a quasi‐parallel shock. We analyze one crossing of Earth's bow shock by the four MMS spacecraft where a number of SLAMS are observed. We find that the transition from upstream to downstream takes place in the antisunward direction, indicating that a SLAMS is impacting the bow shock, causing it to reform. We demonstrate this process by inserting three virtual spacecraft into a hybrid‐Vlasov bow shock simulation. In the simulation, we observe a SLAMS forming ∼30*d*_*i*,*u*_ upstream of the shock and later impacting the shock. During this process, the virtual spacecraft record the same multi‐spacecraft signatures during the shock crossing, thus showing that this reformation process recreates the MMS observations. We propose that the combination of what, in single spacecraft observations, looks like an upstream‐to‐downstream shock crossing, but with the multi‐spacecraft observations in the reverse order, is a clear sign of reformation and can be used in the future to identify these events.

These findings advance our understanding of the cyclical nature of collisionless shocks. Liu et al. (2021) found that periodic changes in the upstream conditions due to ULF waves can modulate the oblique shock and cause it to reform. In this work, we observe reformation at a moderately high‐Mach number quasi‐parallel shock. Under these conditions, the observations and simulation show that shock‐like SLAMS form in the upstream and merge with the shock, causing reformation. The simulation shows that the SLAMS and the shock coexist for some time before reformation, similar to recent observations of the quasi‐perpendicular bow shock (Madanian et al., 2021). The MMS observations and the simulation also indicate that SLAMS form rather close to the shock, in agreement with findings by Lucek et al. (2008) and that SLAMS are an integral part of the reformation process at quasi‐parallel shocks. Future studies should focus on the role of the shock reformation in particle heating and energization at quasi‐parallel shocks.

## Supporting information

Supporting Information S1

Movie S1

## Data Availability

The MMS data are available through the MMS Science Data Center https://lasp.colorado.edu/mms/sdc/public/, and the CDA Web https://cdaweb.sci.gsfc.nasa.gov/index.html. The OMNI data are available from the GSFC/SPDF OMNIWeb interface https://omniweb.gsfc.nasa.gov. Vlasiator (Pfau‐Kempf et al., 2021) is distributed under the GPL‐2 open‐source license. The run described here take several terabytes of disk space and are kept in storage maintained within the CSC‐IT Center for Science. Vlasiator data presented in this paper can be accessed by following the data policy https://www2.helsinki.fi/en/researchgroups/vlasiator/rules-of-the-road.
