# Supplementary material for: Quasi‐Parallel Shock Reformation Seen by Magnetospheric Multiscale and Ion‐Kinetic Simulations
Source: Geophys Res Lett. 2022 Jan 25;49(2):e2021GL096335. doi: 10.1029/2021GL096335 (PMC9285775; doi:10.1029/2021GL096335)
Supplement: Supplementary file 1 — Supporting Information S1 [file GRL-49-0-s001.pdf]

Supporting Information for ”Quasi-parallel Shock Reformation Seen by Magnetospheric Multiscale and Ion-kinetic Simulations”

A. Johlander[1,2], M. Battarbee[2], L. Turc[2], U. Ganse[2], Y. Pfau-Kempf[2], M. Grandin[2], J. Suni[2], V. Tarvus[2], M. Bussov[2], H. Zhou[2], M. Alho[2], M. Dubart[2], H. George[2], K. Papadakis[2], M. Palmroth[2,3]

[1]Swedish Institute of Space Physics, Uppsala, Sweden

[2]Department of Physics, University of Helsinki, Helsinki, Finland

[3]Finnish Meteorological Institute, Helsinki, Finland

Additional Supporting Information (Files uploaded separately)

1. Supporting animation S1.

Introduction

The supporting information consists of one animation showing the simulation run described in the paper.

Movie S1.

Animation of the simulation. Left: the quasi-parallel bow shock. Right: zoom-in on the black box in left panel. The virtual spacecraft VS1-3 are shown as colored dots. Black (blue) contour line shows where B (N_i) is 2 times the upstream value.
